# A Novel Trichothecene Toxin Phenotype Associated with Horizontal Gene Transfer and a Change in Gene Function in *Fusarium*

**DOI:** 10.3390/toxins15010012

**Published:** 2022-12-24

**Authors:** Robert H. Proctor, Guixia Hao, Hye-Seon Kim, Briana K. Whitaker, Imane Laraba, Martha M. Vaughan, Susan P. McCormick

**Affiliations:** 1 Mycotoxin Prevention and Applied Microbiology, National Center for Agricultural Utilization Research, Agricultural Research Service, US Department of Agriculture, 1815 N University St., Peoria, IL 61604, USA; 2 Oak Ridge Institute for Science and Education, Mycotoxin Prevention and Applied Microbiology, National Center for Agricultural Utilization Research, Agricultural Research Service, US Department of Agriculture, Peoria, IL 61604, USA

**Keywords:** trichothecene, mycotoxin, secondary metabolite, *Fusarium*, biosynthetic gene cluster, horizontal gene transfer

## Abstract

*Fusarium* trichothecenes are among the mycotoxins of most concern to food and feed safety. Production of these mycotoxins and presence of the trichothecene biosynthetic gene (*TRI*) cluster have been confirmed in only two multispecies lineages of *Fusarium*: the *Fusarium incarnatum*-*equiseti* (Incarnatum) and *F. sambucinum* (Sambucinum) species complexes. Here, we identified and characterized a *TRI* cluster in a species that has not been formally described and is represented by *Fusarium* sp. NRRL 66739. This fungus is reported to be a member of a third *Fusarium* lineage: the *F. buharicum* species complex. Cultures of NRRL 66739 accumulated only two trichothecenes, 7-hydroxyisotrichodermin and 7-hydroxyisotrichodermol. Although these are not novel trichothecenes, the production profile of NRRL 66739 is novel, because in previous reports 7-hydroxyisotrichodermin and 7-hydroxyisotrichodermol were components of mixtures of 6–8 trichothecenes produced by several *Fusarium* species in Sambucinum. Heterologous expression analysis indicated that the *TRI13* gene in NRRL 66739 confers trichothecene 7-hydroxylation. This contrasts the trichothecene 4-hydroxylation function of *TRI13* in other *Fusarium* species. Phylogenetic analyses suggest that NRRL 66739 acquired the *TRI* cluster via horizontal gene transfer from a close relative of Incarnatum and Sambucinum. These findings provide insights into evolutionary processes that have shaped the distribution of trichothecene production among *Fusarium* species and the structural diversity of the toxins.

## 1. Introduction

**Figure 1:**
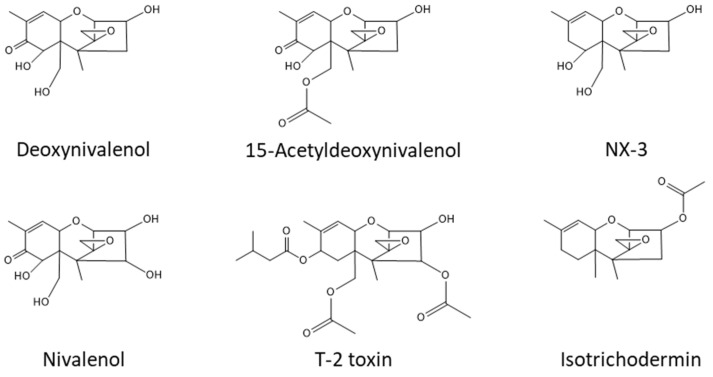
Chemical structures of selected trichothecene analogs produced by *Fusarium* species.

The fungus *Fusarium* poses a dual threat to agriculture by causing crop diseases and contaminating infected crops with mycotoxins such as trichothecenes. Some *Fusarium* trichothecene analogs are among the mycotoxins of most concern to food and feed safety. DNA-based phylogenetic analyses have resolved *Fusarium* into 23 multi-species lineages, which are referred to as species complexes [1,2]. Although the literature has preliminary reports of trichothecene production by members of several of these complexes, more thorough analyses using state-of-the-art analytical chemistry and DNA-based taxonomic methods indicate that trichothecene production in *Fusarium* is limited to two closely related complexes: the *F. incarnatum-equiseti* species complex (Incarnatum) and the *F. sambucinum* species complex (Sambucinum) [3,4,5]. Most incidents of trichothecene contamination of crops have been attributed to members of the latter complex [6]. Collectively, members of Incarnatum and Sambucinum produce structurally diverse trichothecene analogs that vary in the presence and types of oxygen-containing substituents at carbon atoms 3, 4, 7, 8 and 15 (C3, C4, C7, C8 and C15, respectively) of the core trichothecene structure, 12,13-epoxytrichothec-9-ene (EPT) (Figure 1).

Members of Incarnatum and Sambucinum have 13–16 known trichothecene biosynthetic (*TRI*) genes. In Sambucinum, *TRI* genes occur at three loci [7,8,9]. The first locus is the *TRI* cluster, which consists of 10–12 genes. Two of the cluster genes (*TRI4* and *TRI5*) encode enzymes that catalyze reactions resulting in formation of isotrichodermol (3-hydroxy EPT). Five other cluster genes encode enzymes that catalyze hydroxylation (*TRI11* and *TRI13*), acetylation (*TRI3* and *TRI7*) or deacetylation (*TRI8*) during trichothecene biosynthesis. The cluster also includes transcriptional regulatory genes (*TRI6* and *TRI10*), a trichothecene transporter gene (*TRI12*), and two genes of unknown function (*TRI9* and *TRI14*). The second Sambucinum *TRI* locus has only one *TRI* gene, *TRI101*, which encodes an acetyltransferase that catalyzes acetylation of the hydroxyl at C3 [10,11]. The third Sambucinum *TRI* locus can consist of two *TRI* genes, *TRI1* and *TRI16*, but *TRI16* is pseudogenized or absent in some species [12]. *TRI1* encodes a cytochrome P450 monooxygenase that catalyzes oxygenation of C7 and/or C8, and *TRI16* encodes an acyltransferase that catalyzes acylation of the oxygen atom at C8 [7,13]. Collectively, members of Incarnatum have the same complement of *TRI* genes as members of Sambucinum, but *TRI1* and *TRI101* are located in the Incarnatum *TRI* cluster rather than at other loci [5,12]. In addition, the Incarnatum *TRI* cluster includes a third transcriptional regulatory gene, *TRI21* [14]. Variation in trichothecene structures produced by different *Fusarium* species or different strains of the same species results from variable functions of some *TRI* genes (*TRI1* and *TRI8*) and/or variation in the presence of functional alleles of some genes (*TRI1*, *TRI7*, *TRI13*, *TRI16*). The occurrence of the *TRI* cluster in Incarnatum and Sambucinum has been attributed to the presence of the cluster in a common ancestor of these two closely related species complexes and subsequent vertical inheritance of the cluster as the two complexes diverged from one another [15].

During a study of species diversity in the *Fusarium buharicum* species complex (Buharicum), *TRI* genes were detected in the genome sequence of *Fusarium* sp. NRRL 66739, but the report provided no details as to which *TRI* genes were detected [16]. Hereafter, *Fusarium* sp. NRRL 66739 will be abbreviated as 66739. Maximum likelihood and maximum parsimony analyses of partial sequences of four loci (*TEF1*, *RPB1*, *RPB2*, and ITS rDNA) resolved members of Buharicum into seven species: *F. abutilonis*, *F. buharicum*, *F. convolutans*, *F. guadeloupense*, *F. sublunatum* and two undescribed species [16]. 66739 was the sole representative of one of the undescribed species. Detection of *TRI* genes in the genome sequence of 66739 was notable because, to our knowledge, trichothecene production has not been reported in any member of Buharicum. Thus, the current study was initiated to assess: (i) how 66739 *TRI* loci differ from *TRI* loci in other trichothecene-producing *Fusarium* species; (ii) whether 66739 produces trichothecenes; and (iii) the evolutionary relationships of *TRI* genes in 66739 and members of Incarnatum and Sambucinum. Our results provide evidence that horizontal gene transfer (HGT) of part of the *TRI* cluster and a change in function of a transferred gene have contributed to a novel trichothecene production phenotype in 66739.

## 2. Results

For the purposes of this study, the *F. buharicum*, *F. incarnatum*-*equiseti* and *F. sambucinum* species complexes are abbreviated as Buharicum, Incarnatum and Sambucinum, respectively. For clarity, we note that the abbreviation Sambucinum (regular type) used for the *F. sambucinum* species complex [5,17] is distinct from both the *Sambucinum* Clade within the species complex and the species *F. sambucinum* within the *Sambucinum* Clade [3].

### 2.1. TRI Genes in 66739

**Figure 2:**
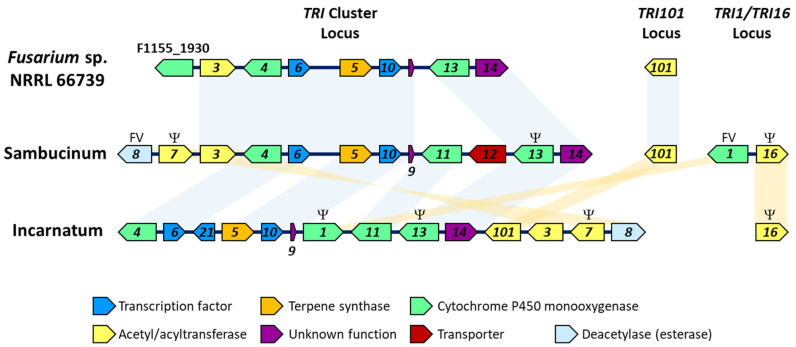
Comparison of the content and organization of genes at trichothecene biosynthetic (*TRI*) loci in *Fusarium* sp. NRRL 66739 and members of the *F. incarnatum-equiseti* (Incarnatum) and *F. sambucinum* (Sambucinum) species complexes. Genes are represented by arrows that point in the direction of transcription. Numbers within arrows indicate specific *TRI* genes (e.g., *4* indicates *TRI4*). Different arrow colors indicate general categories of gene function as defined in the key at the bottom of the figure. The letters FV above an arrow indicate that the function of the corresponding gene can vary among and/or within *Fusarium* species. The Greek letter Psi (Ψ) above an arrow indicates that the gene can be nonfunctional, pseudogenized or completely absent in some species or some strains within species. The blue and orange shading highlight similarity of arrangements of *TRI* gene homologs among the species complexes.

In BLASTn analysis, sequences of homologs of 16 known *TRI* genes from *Fusarium sporotrichioides* and *F. scirpi* were used to query a genome sequence assembly of 66739. This analysis revealed the presence of nine full-length *TRI* gene distributed on two contigs in the 66739 assembly. The first contig, c82, had eight *TRI* genes (*TRI3*, *TRI4*, *TRI5*, *TRI6*, *TRI9*, *TRI10*, *TRI13* and *TRI14*) arranged contiguously in a manner similar to their arrangements in *TRI* cluster homologs in members of Incarnatum and Sambucinum, although the arrangement in 66739 was more similar to the arrangement in Sambucinum (Figure 2). The second 66739 contig, c929, had one *TRI* gene, *TRI101*. In the BLASTn analysis of the 66739 assembly, homologs of the following *Fusarium TRI* genes were not detected: *TRI1*, *TRI7*, *TRI8*, *TRI11*, *TRI12*, *TRI16* and *TRI21*. Because *TRI* cluster homologs have been reported only in Incarnatum and Sambucinum [6], the presence of the *TRI* cluster genes in a member of Buharicum (i.e., 66739) was unexpected. In contrast to the *TRI* cluster, *TRI101* homologs have been detected in all *Fusarium* species that have been examined, including species that do not produce trichothecenes [12,18,19]. Therefore, detection of *TRI101* in 66739 was expected.

We used sequences of *F. sporotrichioides*, *F. scirpi* and 66739 *TRI* genes as queries in BLASTn analysis of genome sequence assemblies of six other members of Buharicum: *F. abutilonis* NRRL 66737, *F. buharicum* NRRL 13371, *F. guadeloupense* NRRL 36125 and NRRL 66743, *F. sublunatum* NRRL 13384, and *Fusarium* sp. NRRL 66182. *TRI101* was detected in all six assemblies, and a 327-base fragment of *TRI13* and a full-length *TRI14* were detected in the *F. sublunatum* assembly. However, the *TRI14* sequence included two single-nucleotide substitutions that introduced premature stop codons at positions 892 and 1097 of the 1113-base coding region that likely rendered the gene nonfunctional. No *TRI* genes other than *TRI101* were detected in the other genome sequence assemblies of Buharicum.

### 2.2. Trichothecene Production by 66739

**Figure 3:**
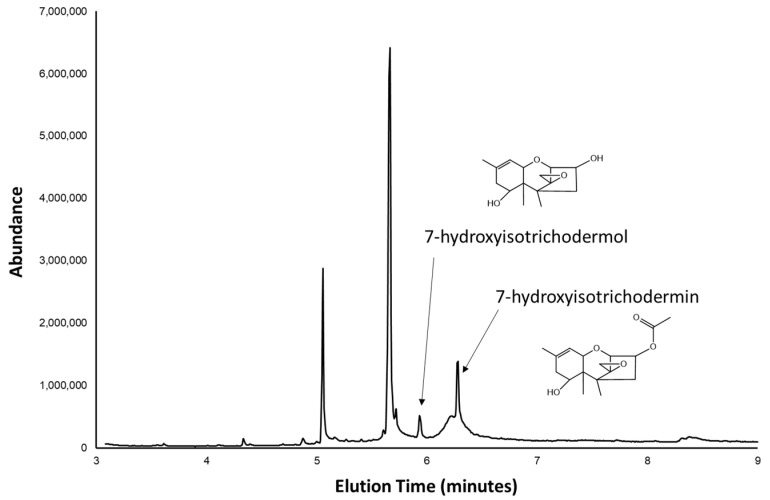
Total ion chromatogram from gas chromatography-mass spectrometry analysis of an ethyl acetate extract of a culture of *Fusarium* sp. NRRL 66739 grown on autoclaved rice kernels.

We assessed trichothecene production in 66739 by growing the fungus in/on three growth media: yeast-peptone-dextrose medium (YEPD), agmatine medium, and rice kernel medium. Thin layer chromatography (TLC) with the reagents 4-(*p*-nitrobenzyl)pyridine (NBP) and tetraethylenepentamine (TEPA) was used to assess the presence of epoxide-containing compounds, including trichothecenes. No epoxide-containing compounds were detected in ethyl acetate extracts of liquid cultures grown in YEPD or agmatine medium, but TLC of extracts of cultures grown on autoclaved rice yielded two blue spots indicative of epoxides and presumably trichothecenes. Gas chromatography-mass spectrometry (GCMS) analysis of the culture extracts confirmed the results of the TLC. That is, GCMS analysis of the extracts of the liquid YEPD and agmatine medium cultures provided no evidence of the presence of trichothecenes. However, GCMS analysis of the autoclaved rice culture extracts indicated the presence of two trichothecene analogs (Figure 3). The trichothecene analogs were identified on the basis of their mass spectra as 7-hydroxyisotrichodermin (6.3 min elution time) and 7-hydroxyisotrichodermol (5.8 min elution time). The structures were confirmed by NMR spectroscopy and by GCMS comparisons with 4-hydroxyisotrichodermin, 8-hydroxyisotrihcodermin, and 15-hydroxyisotrichodermin (data not shown). Both of the trichothecene analogs detected in 66739 rice cultures have been reported in cultures of *Fusarium* species in Sambucinum [20,21,22].

Mass spectral data for 7-hydroxyisotrichodermin and 7-hydroxyisotrichodermol is described below. 7-hydroxylisotrichodermin—retention time 6.3 min; EI mass spectrum, *m/z* (relative intensity): 109 (100), 123 (49), 139 (58), 140 (58), 159 (25), 177 (27), 218 (15), 308 (2). 7-hydroxylisotrichodermol—retention time 5.9 min; EI mass spectrum, *m/z* (relative intensity): 109 (100), 123 (40), 139 (52), 140 (37), 159 (10), 218 (5), 266 (0.3).

### 2.3. 66739 TRI13 Confers Trichothecene 7-Hydroxylation

**Figure 4:**
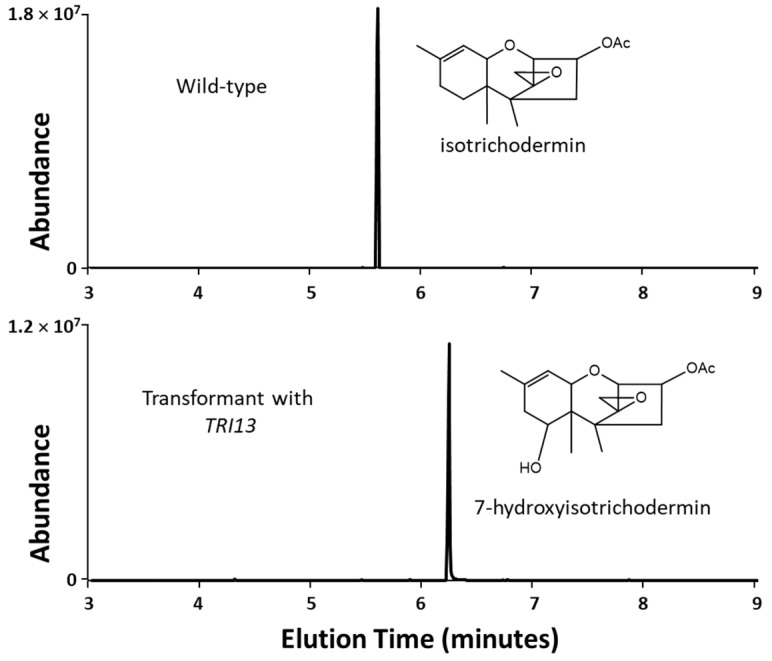
Ability of a *Fusarium verticillioides* transformant to modify the trichothecene biosynthetic intermediate isotrichodermin. In the experiments, isotrichodermin was added to cultures of wild-type *F. verticillioides* (**top**) and a transformant of the fungus engineered to express the *TRI13* homolog from *Fusarium* sp. NRRL 66739 (**bottom**).

In *Fusarium* species that have been examined previously, trichothecene 7-hydroxylation is catalyzed by a cytochrome P450 monooxygenase encoded by the gene *TRI1* [23,24]. Because the genome sequence of 66739 lacks a *TRI1* homolog, the fungus must have another gene that confers 7-hydroxylation. We hypothesized that the 7-hydroxylation gene in 66739 was *TRI13* or F1155_1930, because both of these genes encode a cytochrome P450 monooxygenase, a class of enzymes that often have hydroxylase activity, and the two genes are located within or adjacent to, respectively, the *TRI* cluster in 66739 (Figure 2). To test this hypothesis, we expressed the *TRI13* and F1155_1930 homologs from 66739 in *Fusarium verticillioides*, a species that lacks *TRI* genes, except for *TRI101*, and then fed the trichothecene biosynthetic intermediate isotrichodermin (3-acetyl EPT) to selected transformants carrying either of the two heterologously expressed genes. GCMS analysis of the selected *F. verticillioides* transformants carrying 66739 *TRI13* indicated that they converted isotrichodermin to 7-hydroxyisotrichodermin (Figure 4). In contrast, wild-type *F. verticillioides* and transformants carrying 66739 F1155_1930 did not modify isotrichodermin. These results indicate that the 66739 *TRI13* confers trichothecene 7-hydroxylation. The experiments provided no evidence that 66739 *TRI13* conferred hydroxylation of isotrichodermin at other positions.

### 2.4. Phylogenetic Relationships of 66739 to Other Fusarium Species

**Figure 5:**
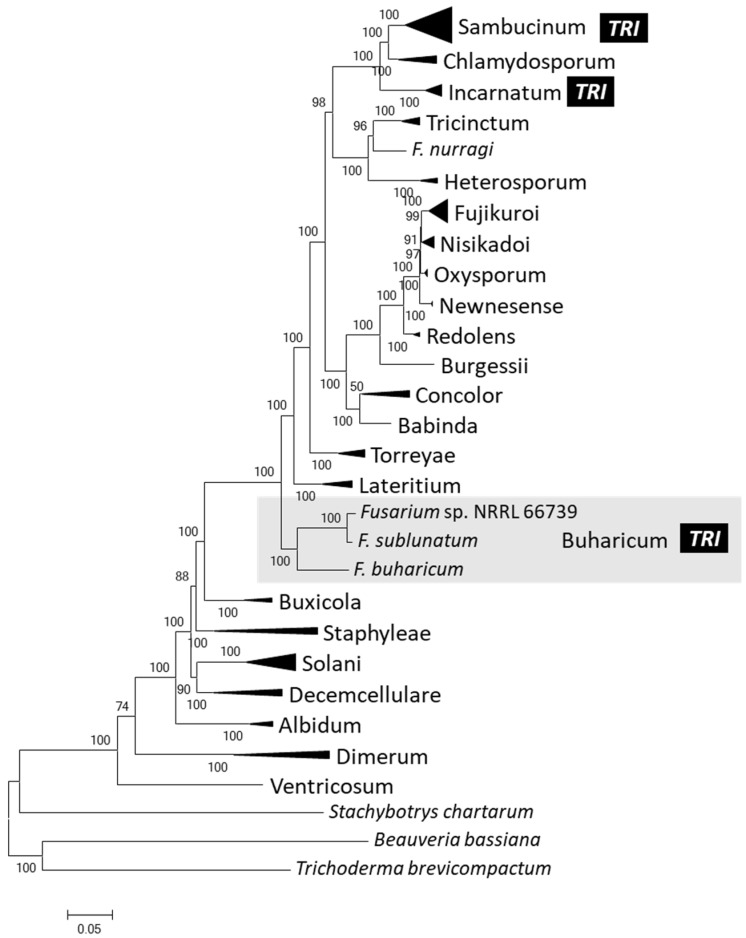
Maximum likelihood tree of selected species of *Fusarium* showing the 23 previously described species complexes and the monotypic lineage consisting of *F. nurragi*. With the exception of the *F. buharicum* species complex, clades corresponding to species complexes have been collapsed and represented as black triangles so that individual taxa are not shown. The tree was inferred using concatenated alignments of a previously described set of 19 housekeeping genes [1]. Numbers next to branches are bootstrap values generated from 1000 pseudoreplications. Species complex names are abbreviated using the specific epithet of the species after which each complex is named, except that the first letter is uppercase and all letters are in regular type—e.g., the *F. fujikuroi* and *F. sambucinum* species complexes are abbreviated as Fujikuroi and Sambucinum, respectively. Species complexes in which a trichothecene biosynthetic gene cluster homolog has been detected are indicated with 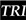.

To confirm that 66739 is a member of Buharicum, we inferred a species tree that included 66739, two other members of Buharicum (*F. buharicum* and *F. sublunatum*), and 82 other species representing the other 22 *Fusarium* species complexes that have been described. The tree was inferred from aligned DNA sequences of full-length coding regions of a previously described set of 19 housekeeping genes [1]. In 15 of the 19 individual gene trees, 66739, *F. sublunatum* and *F. buharicum* were resolved as an exclusive and well-supported clade (bootstrap values = 92 to 100). In all 19 individual gene trees, 66739 and *F. sublunatum* formed an exclusive and well-supported clade (bootstrap values = 100) (Figure S1). In a tree inferred from concatenated alignments of all 19 genes, 66739, *F. buharicum* and *F. sublunatum* were resolved as an exclusive clade with a bootstrap value of 100 (Figure 5). Thus, our results confirm the previous report that assigned 66739 to Buharicum [16]. In the 19-gene species tree, most of the other 22 species complexes were also resolved as exclusive and well-supported clades (bootstrap values = 91 to 100). The one exception was the *F. concolor* species complex, which did not have significant bootstrap support in the tree inferred from the concatenated alignments (Figure 5). Buharicum was located near the base of the *Fusarium* species tree, while Incarnatum and Sambucinum were located near one another at the top of the tree (Figure 5). These results confirm that 66739 is distantly related to members of Incarnatum and Sambucinum, which are the only other *Fusarium* species in which *TRI* cluster homologs have been identified and trichothecene production has been confirmed [6].

### 2.5. Assessment of Potential Horizontal Transfer of TRI Cluster

**Figure 6:**
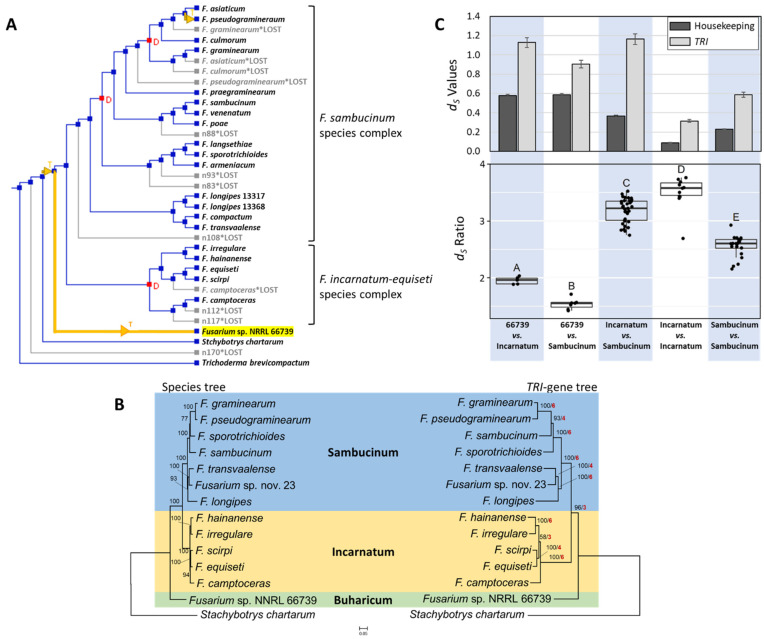
Results of phylogenetic assessments of horizontal gene transfer (HGT) of *TRI* genes. (**A**)—Results of NOTUNG analysis. Inference of HGT of *TRI* genes to *Fusarium* sp. NRRL 66739 (highlighted in yellow) from a common ancestor of the *Fusarium incarnatum*-*equiseti* (Incarnatum) and *F. sambucinum* (Sambucinum) species complexes is indicated by the orange branch with the arrow marked with T in orange type. The tree and species names were from an output file from NOTUNG analysis. Some information in the original NOTUNG figure (e.g., species names) was redrawn for clarity. Other inferences made in the tree (HGT events indicated by orange branches with an arrow, duplications indicated by red nodes labelled D, and loss events indicated by grey branches and *LOST) are not discussed here. (**B**)—Manual comparison of a species tree (left) and *TRI*-gene tree (right). Numbers in black type next to branches are bootstrap values generated from 1000 pseudoreplications. In the *TRI*-gene tree, numbers in red type next to branches are gene concordance values. (**C**)—Summaries of analyses of estimates of synonymous changes per synonymous site (*d_S_*). **Top**—mean *d_S_* values of housekeeping genes and *TRI* genes for all possible pairwise comparisons of species within and among species complexes. Error bars are means of standard errors obtained from the *d_S_* analysis. **Bottom**—ratios of *TRI* gene *d_S_* values to housekeeping gene *d_S_* values (*d_S_* ratios). Boxplots indicate the median (thick horizontal line), 25th and 75th percentiles (box), and range of the data. Each point represents a ratio derived from one pairwise comparison within a treatment (indicated by alternate blue and white shading). Different letters above the boxes denote statistically significant differences among treatments. The species used in these analyses were the same as those shown in Figure 6B except that *Stachybotrys chartarum* was excluded.

Horizontal gene transfer (HGT) is a possible explanation for the presence of the *TRI* cluster in distantly related *Fusarium* species complexes and its absence in intervening lineages (Figure 5). To assess whether there is evidence to support HGT of the cluster, we did a series of phylogenetic analyses that have been used previously to assess the likelihood of HGT [5,25,26]. In the first analysis, we used the program NOTUNG, which reconciles differences between species trees and gene trees by inferring HGT, gene duplication, or lineage sorting. In our NOTUNG analysis, the species tree consisted of the *Fusarium* species tree used to prepare Figure 5. The gene tree consisted of a tree inferred from concatenated alignments of six *TRI*-cluster genes (*TRI3*, *TRI4*, *TRI5*, *TRI6*, *TRI10* and *TRI14*) that are common to a subset of fungi in the species tree: 66739, selected members of Incarnatum and Sambucinum, *Stachybotrys chartarum* and *Trichoderma brevicompactum* (Figure S2). The results of the NOTUNG analysis included inference of HGT of the *TRI* genes from an ancestor of Incarnatum and Sambucinum to 66739 (Figure 6A).

In a second analysis, we conducted a manual comparison of the topologies of a species and *TRI*-gene tree that included 66739, 12 species representing a range of phylogenetic diversity within Incarnatum and Sambucinum, and *S. chartarum* as an outgroup. The species tree was inferred from concatenated alignments of the 19 housekeeping genes noted above, and the *TRI* tree was inferred from concatenated alignments of the *TRI3*, *TRI4*, *TRI5*, *TRI6*, *TRI10* and *TRI14* coding regions. The topology of the resulting *TRI* gene tree mirrored the species tree with respect to 66739, Incarnatum and Sambucinum (Figure 6B). However, this result was ambiguous with respect to HGT because the *Fusarium TRI* gene sequences used in the analysis were from members of Incarnatum and Sambucinum and 66739, but the NOTUNG-inferred HGT involved transfer from an Incarnatum-Sambucinum ancestor to 66739. As a result, the topology of the *TRI* gene tree in Figure 6B could result from 66739 acquiring *TRI* genes by HGT from an ancestor of Incarnatum and Sambucinum or by vertical inheritance from a common ancestor of Buharicum, Incarnatum and Sambucinum.

In a third analysis, we compared divergence of *TRI* genes versus housekeeping genes using estimates of synonymous changes per synonymous site (*d_S_*). This analysis was based on the following suppositions: (i) levels of divergence of gene homologs should be positively correlated with duration of divergence; and (ii) in comparisons of two organisms, divergence times should be longer for vertically inherited genes than for horizontally transferred genes, because vertically inherited genes would have been diverging since the organisms diverged from their common ancestor, while horizontally transferred genes would have been diverging since the HGT event. Thus, we expected divergence levels of vertically inherited genes to be greater than those of horizontally transferred genes [5,26]. In pairwise comparisons of 66739 with members of Incarnatum or Sambucinum, *TRI* genes had consistently higher levels of divergence than housekeeping genes. This is the opposite of what we expected if the NOTUNG-inferred HGT event had occurred (Figure 6C, upper panel). Thus, taken at face value, the *d_S_* values were more consistent with 66739 acquiring the *TRI* cluster by vertical inheritance from a common ancestor of Buharicum, Incarnatum and Sambucinum than by HGT from a common ancestor of Incarnatum and Sambucinum.

However, the *TRI* gene *d_S_* values were also consistently higher than housekeeping gene *d_S_* values in comparisons between and within Incarnatum and Sambucinum. In other words, *Fusarium* lineages in which *TRI* genes are thought to be vertically inherited had consistently higher *d_S_* values for *TRI* than housekeeping genes (Figure 6C Top). The higher *TRI* gene *d_S_* values were consistent with previous reports that vertically inherited secondary metabolite biosynthetic (SMB) genes tend to diverge more rapidly than vertically inherited housekeeping genes [5,17]. This observation led us to a fourth analysis in which we examined ratios of *TRI*-gene *d_S_* values to housekeeping-gene *d_S_* values (hereafter *d_S_* ratios). The idea behind this fourth analysis was, given the tendency of SMB genes to diverge more rapidly than housekeeping genes, *d_S_* ratios should increase with increasing duration of divergence. That is, if 66739 acquired the *TRI* cluster by vertical inheritance from a common ancestor of Buharicum, Incarnatum and Sambucinum, *d_S_* ratios resulting from comparisons of 66739 versus Incarnatum or Sambucinum (i.e., distantly related taxa) should be greater than ratios from comparisons within and between Incarnatum and Sambucinum (i.e., more closely related taxa). However, the observed *d_S_* ratios were not consistent this expectation. Specifically, *d_S_* ratios for 66739 versus Incarnatum or Sambucinum comparisons were significantly lower (*p* < 0.001) than from all comparisons within and between Incarnatum and Sambucinum (Figure 6C). Given the rationale above, the *d_S_* ratios were not consistent with 66739 acquiring the *TRI* cluster by vertical inheritance from a common ancestor of Buharicum, Incarnatum and Sambucinum.

## 3. Discussion

The finding that 66739 produces trichothecenes indicates that production of the mycotoxins occurs more widely in the genus *Fusarium* than previously recognized [5,6,15]. The absence of an intact cluster in the other members of Buharicum examined indicates that the cluster has a limited distribution within the complex and is consistent with a previous report that *F. buharicum* did not produce trichothecenes [27]. However, the presence of a *TRI13* remnant and a *TRI14* pseudogene in *F. sublunatum* indicates that the *TRI* cluster and trichothecene production might have been more widely distributed in Buharicum in the past. By contrast, the cluster is present in all extant members of Incarnatum and Sambucinum that have been examined [6].

### 3.1. Change in TRI13 Function

Our heterologous expression analysis indicated that *TRI13* confers formation of the 7-hydroxyl substituent of trichothecenes produced by 66739. This contrasts evidence indicating that *TRI13* confers trichothecene 4-hydroxylation in Incarnatum and Sambucinum [5,13,28]. Further, in other fusaria that have been examined previously, trichothecene 7-hydroxylation is conferred by the cytochrome P450 monooxygenase gene *TRI1* [23,24]. Together, results from the current and previous studies indicate that the function of *TRI13* has changed during its evolutionary history. This raises the question: Was the ancestral function of *TRI13* 4-hydroxylation or 7-hydroxylation of trichothecenes? Below, we consider two alternative scenarios (A and B) that address this question. Scenario A—the ancestral *TRI13* conferred 4-hydroxylation; this function was retained in Incarnatum and Sambucinum but changed to 7-hydroxylation in 66739; and in Incarnatum and Sambucinum, trichothecene 7-hydroxylation has always been conferred by *TRI1*. Scenario B—the ancestral *TRI13* conferred 7-hydroxylation; this function was retained in 66739 but changed to 4-hydroxylation in a common ancestor of Incarnatum and Sambucinum; and the Incarnatum-Sambucinum ancestor then acquired the ability to hydroxylate trichothecenes at C7 by acquisition or change in function of *TRI1*. Scenario A requires only one change, a change in *TRI13* function, whereas Scenario B requires two changes, a change in *TRI13* function and acquisition (or change in function) of *TRI1*. We reason that because Scenario A is more parsimonious it is more likely. Scenario B cannot be ruled out completely, however, because of evidence that functions of multiple *TRI* genes have changed during their evolutionary history [13,23,24,29].

Together, results from the current and previous studies indicate that trichothecene 7-hydroxylation has evolved independently two times in the genus *Fusarium*. Results from previous studies indicate that 7-hydroxylation evolved once in an ancestor of Incarnatum and Sambucinum as a result of the activity of the enzyme Tri1 [12,23,30] and, assuming Scenario A above is correct, results from the current study indicate that 7-hydroxylation evolved independently in Buharicum as a result of a change in the activity of the enzyme Tri13.

### 3.2. Novel Trichothecene Production Phenotype and Biosynthetic Pathway

**Figure 7:**
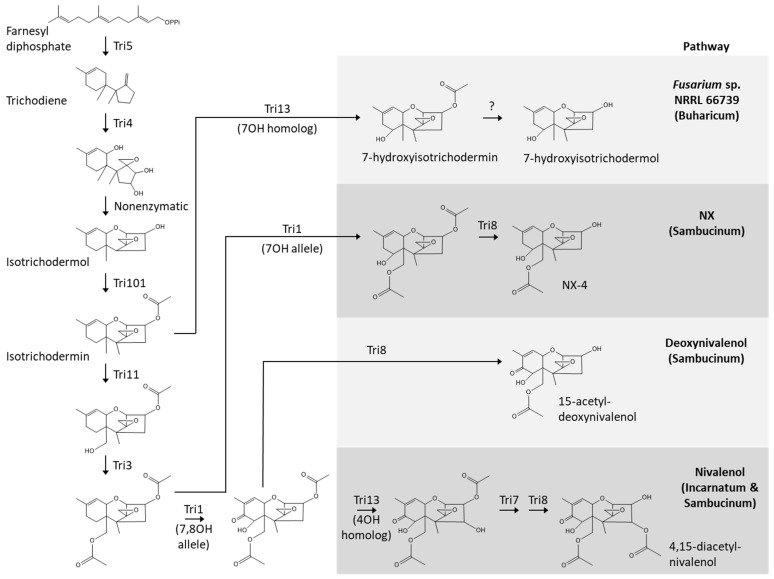
Comparison of the proposed biosynthetic pathway for 7-hydroxyisotrichodermin and 7-hydroxyisotrichodermol in *Fusarium* sp. NRRL 66739 with previously described pathways for NX, deoxynivalenol and nivalenol in the *Fusarium incarnatum-equiseti* (Incarnatum) and *F. sambucinum* (Sambucinum) species complexes. Tri followed by a number indicates the trichothecene biosynthetic enzyme that catalyzes the corresponding reaction.

Production of 7-hydroxyisotrichodermin and 7-hydroxyisotrichodermol by certain members of Sambucinum has been reported previously [20,21,31]. The strains of *Fusarium* examined in the previous reports also produced higher levels of several other trichothecene analogs, such as the acetylated DON and NIV analogs 3-acetyldeoxynivalenol and 4,15-diacetylnivalenol, respectively. Thus, production of only 7-hydroxyisotrichodermin and 7-hydroxyisotrichodermol by 66739 constitutes a novel trichothecene production phenotype. The presence of only eight genes in the 66739 *TRI* cluster compared to the 10–14 genes in the Incarnatum and Sambucinum *TRI* clusters is consistent with the relative structural simplicity of 7-hydroxyisotrichodermin and 7-hydroxyisotrichodermol relative to some trichothecene mycotoxins produced by members of Incarnatum and Sambucinum [3,32] (Figure 1). Based on the roles of *TRI* genes determined in previous studies [7], the role of *TRI13* reported in the current study, and the *TRI*-gene content in 66739, we have proposed a biosynthetic pathway for 7-hydroxyisotrichodermin and 7-hydroxyisotrichodermol in 66739 (Figure 7). The first four steps of the proposed pathway are the same as those in the DON, NIV and NX pathways. The proposed 66739 pathway diverges from the other pathways with the Tri13-catalyzed 7-hydroxylation of isotrichodermin to form 7-hydroxyisotrichodermin. The final step of the proposed pathway is 3-deacetylation of 7-hydroxyisotrichodermin to form 7-hydroxyisotrichodermol. It is not clear what gene confers this last biosynthetic step, because 66739 lacks the gene (*TRI8*) that confers 3-deacetylation in other fusaria [9]. Overall, the proposed pathway in 66739 requires fewer biosynthetic reactions than the DON, NIV and NX pathways, reflecting the relatively simple structures of 7-hydroxyisotrichodermin and 7-hydroxyisotrichodermol (Figure 7).

It is notable that the 66739 cluster includes *TRI3*, which in other fusaria confers trichothecene 15-*O*-acetylation [33]. The function of *TRI3* in 66739 is not clear because synthesis of 7-hydroxyisotrichodermin and 7-hydroxyisotrichodermol does not require 15-*O*-acetylation. Furthermore, 66739 lacks a *TRI11* homolog, which confers 15-hydroxylation; and a 15-hydroxyl is required for 15-*O*-acetylation [13]. It might be possible to determine the role of the 66739 *TRI3* homolog by heterologous expression, in the same manner as the function of *TRI13* was determined in the current study. The results of such an analysis could provide insight into whether 66739 can produce trichothecene analogs in addition to those observed in the current study.

### 3.3. Putative Horizontal Transfer of TRI Cluster

Although the results of the NOTUNG analysis indicate the donor of the putative HGT event was an ancestor of Incarnatum and Sambucinum, the analysis would not be able differentiate this putative donor from a donor that was from an unknown lineage closely related to Incarnatum and Sambucinum. Likewise, the presence of the *TRI13* fragment and *TRI14* pseudogene in *F. sublunatum*, another member of Buharicum, suggests that the recipient of the transfer could have been a common ancestor of 66739 and *F. sublunatum*. Given these considerations, we regard the likely donor of the putative HGT to be an unspecified relative of Incarnatum and Sambucinum (i.e., an ancestor or a species from a related lineage) and the recipient to be an ancestor of 66739.

The multiple assessments of HGT of the *TRI* cluster yielded seemingly conflicting results. Results of the NOTUNG and *d_S_* ratio analyses were consistent with HGT of the cluster, whereas results of analysis of *d_S_* values were consistent with vertical inheritance of the *TRI* cluster from a common ancestor of Buharicum, Incarnatum and Sambucinum. We propose that this apparent conflict resulted from the tendency of SMB genes to diverge more rapidly than housekeeping genes [5,17], which in turn caused fading of the phylogenetic signal for HGT that *d_S_* values can provide. That *Fusarium TRI* genes can diverge more rapidly is evident from the higher *d_S_* values for *TRI* genes than housekeeping genes in comparisons of lineages in which the distribution of the *TRI* cluster is thought to have resulted from vertical inheritance (e.g., in comparisons of Incarnatum versus Sambucinum) (Figure 6C). The following scenario demonstrates how more rapid divergence of *TRI* genes could have caused fading of the phylogenetic signal for HGT. In the scenario, immediately after HGT of the *TRI* cluster, sequences of *TRI* gene homologs in the donor and recipient were the same, but sequences of housekeeping gene homologs were different because they had been diverging since the time the donor and recipient diverged from one another. As a result, the *d_S_* ratio immediately after the transfer would have been very low. As time passed, the *TRI* genes diverged more rapidly than housekeeping genes and, at some point, the divergence levels for *TRI* genes and housekeeping genes became the same in descendants of the donor and recipient, resulting in *d_S_* ratios of 1. As more time passed, the divergence levels of the more rapidly diverging *TRI* genes surpassed those of the housekeeping genes, resulting in *d_S_* ratios of greater than one. Thus, as the duration of time since the HGT event increased, divergence levels and, therefore, *d_S_* values of *TRI* genes relative to housekeeping genes increased, which in turn resulted in fading of the phylogenetic signal provided by the *d_S_* values. We propose that the *TRI* cluster homologs in 66739, Incarnatum and Sambucinum are currently at the latter time point in the scenario described above.

Despite the proposed fading of phylogenetic signal for HGT, we also propose that some signal was retained in the *d_S_* ratios. Our rationale for this is that even though horizontally transferred *TRI* genes diverged more rapidly than housekeeping genes they did not diverge as much as they would have if they had been vertically inherited from a common ancestor of Buharicum, Incarnatum and Sambucinum. This rationale would explain why the *d_S_* ratios for comparisons of 66739 versus Incarnatum or Sambucinum are lower than for the other comparisons. Comparisons of *d_S_* values and *d_S_* ratios for other SMB genes for which there is evidence of HGT should provide additional information on the value of using *d_S_* ratios to detect ancient HGT events.

## 4. Conclusions

The finding that a member of Buharicum can produce trichothecenes indicates that production of the mycotoxins occurs more widely among lineages of *Fusarium* than was previously recognized [6,34]. Evidence that this expanded distribution resulted from HGT of the *TRI* cluster adds to a growing body of evidence that HGT has contributed to the current distribution of mycotoxin production among fungi [5,25,26,35].

Evidence that a change in *TRI13* function has resulted in a novel trichothecene production phenotype adds to knowledge of how changes in *TRI* gene content and function have contributed to structural diversity of trichothecenes. Although the evolutionary driver(s) of the structural diversity has not been identified, knowledge of the role of trichothecenes in pathogenesis of *F. graminearum* points to one possibility. Trichothecene production contributes to the ability of the fungus to spread within wheat heads and thereby cause Fusarium head blight [36,37]. However, there are plant glucosyltransferases that confer resistance to the toxins and in turn resistance to head blight [38]. There is also evidence that the glucosyltransferases are substrate specific [39,40]. Based on this information, we propose the following hypothesis: substrate specificity of enzymes that confer resistance to trichothecenes drives structural diversity of the toxins. Implicit in this hypothesis is the idea that when a *Fusarium* strain acquires a *TRI*-gene mutation that causes a change in trichothecene structure the mutation could allow the strain to evade a substrate-specific enzyme that confers resistance to trichothecenes produced prior to the mutation. Experiments aimed at testing this hypothesis could provide insights into development of enzymes with enhanced activity against trichothecenes.

## 5. Materials and Methods

### 5.1. Abbreviations, Strains and Culture Media

Relatively little information is available on the origins of 66739. It was isolated in China, accessioned into the *Fusarium* Research Center culture collection as FRC L-0453, and subsequently accessioned in the ARS Culture Collection as NRRL 66739 [16]. *Fusarium verticillioides* strain FRC M-3125 was used for heterologous gene expression. Both *Fusarium* strains were stored in glycerol solution at −80 °C and cultured on V8 Juice agar medium [41] for production of conidia and/or mycelia. Liquid agmatine medium, liquid yeast extract peptone dextrose medium (YEPD), and solid rice kernel medium (4.4 g rice kernels and 1.8 mL water) were used for trichothecene production assays [3,42], and liquid glucose-yeast extract-peptone medium (GYP) was used for preparation of genomic DNA [17]. Larger rice cultures, prepared with 440 g rice kernels and 180 mL water, were used for production of sufficient quantities of trichothecene analogs to confirm chemical structures.

### 5.2. In Silico DNA Sequence and Phylogenetic Analyses

Genome sequences of 66739 and six other members of Buharicum were generated in previous studies [1,16,17]. The strains and the corresponding GenBank accessions, in parentheses are as follows: *F. abutilonis* NRRL 66737 (JAJJWN000000000), *F. buharicum* NRRL 13371 (JAATHB000000000), *F. guadeloupense* NRRL 36125 (JAJJWL000000000) and NRRL 66743 (JAJJWM000000000), *F. sublunatum* NRRL 13384 (JABFFF000000000), *Fusarium* sp. NRRL 66182 (JABFAK000000000), and *Fusarium* sp. NRRL 66739 (JAJJWO000000000). BLASTn analyses were done using an in-house database of the genome sequences maintained in CLC Genomics Workbench (Qiagen; Hilden, Germany). *TRI* gene sequences from *F. sporotrichioides* strain NRRL 3299 and *F. scirpi* strain NRRL 66328 used as query sequences in the BLASTn analysis have been described previously [5,28,29]. Sequences identified by BLAST analysis in CLC were analyzed and visualized in Sequencer (https://www.genecodes.com; accessed on 23 December 2022) and/or MEGA7 [43].

For most species, housekeeping genes sequences used to infer species trees were available in the TreeBASE database (Study S27101; https://www.treebase.org/treebase-web/home.html; accessed on 23 December 2022) [1]. For some members of Buharicum, housekeeping gene sequences were retrieved by BLAST analyses from the in-house database noted above. When necessary, gene sequences used herein were manually annotated using MEGA7 [43]. Alignments of gene sequences were done with MUSCLE as implemented in MEGA7 or MAFFT using the L-INS-I alignment method [44]. For some analyses, alignments of individual gene sequences were concatenated using Sequence Matrix [45]. Maximum likelihood trees were then inferred from sequence alignments using IQ-Tree version 1.6.7 [46]. Statistical support for branches in the resulting trees was determined using ultrafast bootstrap analysis in IQ-Tree and, in some cases, gene concordance factors as determined by IQ-Tree [47]. In addition to the tree inference method described above, two other methods were used to assess horizontal gene transfer (HGT): (i) NOTUNG analysis [48]; and estimates of synonymous substitutions per synonymous site (*d_S_* values) calculated with the program CODEML [49]. Statistical analysis of *d_S_* ratios was performed in R (v.4.2.1) [50] using the Estimated Marginal Means method [51] and the package emmeans (v.1.8.1.1) to account for unbalanced design [52]. All pairwise contrasts were computed, and statistical significance adjusted for n = 10 total comparisons using the Bonferroni method.

### 5.3. Trichothecene Analysis

For analysis of trichothecene production, a 0.5 cm^2^ mycelial plug from a V8 Juice agar culture of 66739 was transferred to 20 mL of YEPD or 20 mL of agmatine medium in a 50-mL Erlenmeyer flask, or to solid rice kernel medium in a 6-dram vial [3,42]. Liquid cultures were incubated at 28 °C in the dark with shaking at 200 rpm, and rice cultures were incubated at 25 °C in the dark without shaking. After 7 days of incubation, liquid and rice cultures were extracted with 8 mL and 10 mL, respectively, of ethyl acetate for 30 min on a horizontal vortex shaker adjusted to the highest speed. The extracts were transferred to 1-dram vials and dried under a stream of nitrogen. The resulting residues were each suspended in 1 mL of ethyl acetate and then analyzed for the presence of trichothecenes using two methods: (1) separation on thin layer silica gel with methanol/dichloromethane (5:95) and detection of epoxide containing compounds with the two-stage spray reagent 4-(*p*-nitrobenzyl)pyridine (NBP) and tetraethylenepentamine (TEPA) [53,54]; and (2) gas-chromatography-mass spectrometry (GCMS).

The GCMS system consisted of an Agilent 6890 gas chromatograph, fitted with a HP-5MS column (30 m, 0.25 mm, 0.25 μm), coupled to an Agilent 5973-mass spectrometer (Santa Clara, California, USA). The system used helium as a carrier gas, a split ratio of 20:1, and a split flow of 20 mL per min. After sample injection, the following column temperature regime was used: 150 °C for 1 min; increased by 30 °C per min to 280 °C; and 280 °C for 7.7 min. Chromatographic peaks were identified based on comparisons of their mass spectra with a reference library from the National Institute of Standards (NIST), which includes mass spectra of approximately 50 trichothecene analogs, and the mass spectral library described by Savard and Blackwell [55].

Larger rice cultures were extracted with ethyl acetate, the extract concentrated with a rotary evaporator, and the concentrated extract separated with silica gel column chromatography eluted with methanol/dichloromethane (5:95). Column fractions were monitored with GCMS. Fractions were further purified on a silica gel column eluted with diethyl ether/hexane (4:1) and then on a Sephadex LH-20 column eluted with methanol. Structures were confirmed with ^1^H- and ^13^C-nuclear magnetic resonance (NMR) using an Avance 500 MHz Bruker NMR spectrometer (Bruker Biospin, Billerica, USA).

### 5.4. Heterologous Expression of TRI13 and F1155_1930

Because of its effectiveness in previous studies, we used heterologous expression to assess gene function in the current study [56,57]. To generate expression constructs of the cytochrome P450 genes *TRI13* and F1155_1930, the coding region (with introns) of each gene was fused to the *Aureobasidium pullulans TEF1* promoter sequence (*AbTEF1*pro), cloned into the plasmid pRF-GU as described [58], and then transformed into *F. verticillioides* strain FRC M-3125. In this fungus, *AbTEF1pro* was expected to drive constitutive expression of *TRI13* and F1155_1930 [59]. PCR primers used to generate the constructs are listed in Supplementary Table S1. *AbTEF1*pro was amplified from plasmid pTEFEGFP [59] using PCR primers TEF1-3 and TEF1-5-up, which included the sequence GGACTTAAU at its 5′ end to facilitate USER cloning (New England Biolabs, Ipswich, USA). The *TRI13* coding region was amplified from 66739 genomic DNA with PCR primers TRI13-5 and TRI13-3-down, and the F1155_1930 coding region was amplified with primers CPM1-5 and CPM1-3-down. To facilitate fusion of *AbTEF1*pro and coding regions, the 5′ primer for each coding region (i.e., TRI13-5 and CPM1-5) included a 15-base overlap with the 3′ end of *AbTEF1*pro. In addition, the 3′ primer for each coding region (i.e., TRI13-3-down and CPM1-3-down) included the sequence GGGTTTAAU to facilitate USER cloning. To generate fusion constructs, the *AbTEF1*pro and individual coding-region amplicons were employed as a DNA template in a second round of PCR, using primers TEF-5-up and Tri13-3-down to amplify the *AbTEF1*pro-*TRI13* fusion construct, and primers TEF-5-up and CPM1-3-down to amplify the *AbTEF1*pro-F1155_1930 fusion construct. The resulting fusion constructs were cloned separately into plasmid pRF-GU using the USER protocol as previously described (Hao e al. 2019). Briefly, each amplified fusion construct was ligated separately with pRF-GU that had been digested with restriction enzymes *Pac*I and *Nt.Bbc*CI (New England Biolabs). A 2-μL aliquot of each ligation reaction was transformed separately into *Escherichia coli* TOP10 competent cells (Invitrogen, Waltham, USA). The resulting colonies were screened by PCR with primers TEF-5-up and TRI13-3-down (*AbTEF1*pro-*TRI13*) and primers TEF-5-up and CPM1-3-down (*AbTEF1*pro-F1155_1930) to confirm the presence of the fusion constructs, and selected plasmids (*AbTEF1*pro-*TRI13* and *AbTEF1*pro-F1155_1930 expression vectors) were subjected to Sanger sequencing using TEF1-664F, TRI13-399R or CPM1-437R to confirm that fusion construct sequences were correct.

Each expression vector was transformed into *Agrobacterium tumefaciens* strain AGL1 as previously describe [58]. Briefly, the *A. tumefaciens* transformants were grown overnight in Luria Broth [60] supplemented with spectinomycin to an OD_600_ of 0.4. A 200 μL aliquot of the *A. tumefaciens* cell suspension was then mixed with 200 μL of *F. verticillioides* conidia (1 × 10^7^ conidia per mL) harvested from a 7-day-old V8 Juice agar culture. Two hundred μL of the resulting cell-conidia mixture were spread onto a 0.45-μm pore nitrocellulose filter (Whatman-Cytiva, Marlborough, USA) on co-culture medium [61] amended with 200 μM acetosyringone. After incubation at 25 °C for two days, the filters were transferred to V8 Juice agar medium containing 150 μg/mL hygromycin B and 200 μM cefotaxime (Sigma Aldrich Chemical Co., St. Louis, Missouri, USA). Hygromycin B-resistant colonies of *F. verticillioides* were transferred to fresh V8 Juice agar containing 150 μg/mL hygromycin and 200 µg/mL cefotaxime. Genomic DNA was isolated from the resulting cultures and screened by PCR to confirm the presence of the expression constructs. Primers TEF-5-up & Tri13-3-down and TEF-5-up and CPM1-3-down were used to confirm the presence of the *AbTEF1*pro-*TRI13* and *AbTEF1*pro-F1155_1930 constructs, respectively. Three independently acquired *F. verticillioides* transformants for each expression construct were selected for further analysis.

### 5.5. Feeding with F. verticillioides Transformants and Isotrichodermin

Cultures of three types of *F. verticillioides* strains were used in feeding experiments: (1) wild-type strain FRC M-3125; (2) three transformants with the *AbTEF1*pro-*TRI13* expression construct; and (3) three transformants with the *AbTEF1*pro-F1155_1930 construct. For each strain, two 0.5 cm^2^ plugs from a V8 Juice agar culture were added to 20 mL of YEPD in a 50-mL Erlenmeyer flask. One hundred μL of acetone containing 1.5 mg of the trichothecene analog isotrichodermin (3-acetyl EPT) was added to 20-mL cultures of selected *F. verticillioides* transformants carrying either the *TRI13* or F1155_1930 expression constructs (final isotrichodermin concentration was 250 μM). Cultures were incubated at 28 °C in the dark with shaking at 200 rpm. After 7 days, cultures were extracted with 8 mL ethyl acetate as described above. Extracts were concentrated under a stream of nitrogen and then analyzed by GCMS as described above.

## Data Availability

Genome sequences used in this study are available at the GenBank database at the National Center for Biotechnology Information as accessions JAATHB000000000, JABFAK000000000, JABFFF000000000, JAJJWL000000000, JAJJWM000000000, JAJJWN000000000, and JAJJWO000000000. Fusarium sp. NRRL 66739 is available at the ARS Culture Collection (NRRL).

## Supplementary Materials

The following are available online at https://www.mdpi.com/article/10.3390/toxins15010012/s1, Figure S1: Individual gene trees for 19 housekeeping genes inferred by maximum likelihood analysis as implemented in IQ-Tree. Numbers near branches are bootstrap values based on 1000 pseudoreplicates using the ultrafast bootstrap parameter in IQ-Tree. Figure S2: Individual gene trees for six trichothecene biosynthetic (*TRI*) genes inferred by maximum likelihood analysis as implemented in IQ-Tree. Numbers near branches are bootstrap values based on 1000 pseudoreplicates using the ultrafast bootstrap parameter in IQ-Tree. Table S1: Oligonucleotides used in the current study for polymerase chain reactions (PCR) and Sanger sequence analysis.
