# Supplementary material for: A Novel Trichothecene Toxin Phenotype Associated with Horizontal Gene Transfer and a Change in Gene Function in Fusarium
Source: Toxins (Basel). 2022 Dec 24;15(1):12. doi: 10.3390/toxins15010012 (PMC9864338; doi:10.3390/toxins15010012)
Supplement: Supplementary file 1 [file toxins-15-00012-s001.zip › toxins-2065711-supplementary.pdf]

toxins

MDPI

Article

A Novel Trichothecene Toxin Phenotype Associated with Horizontal Gene Transfer and a Change in Gene Function in *Fusarium*

Robert H. Proctor, Guixia Hao, Hye-Seon Kim, Briana K. Whitaker, Imane Laraba, Martha M. Vaughan and Susan P. McCormick

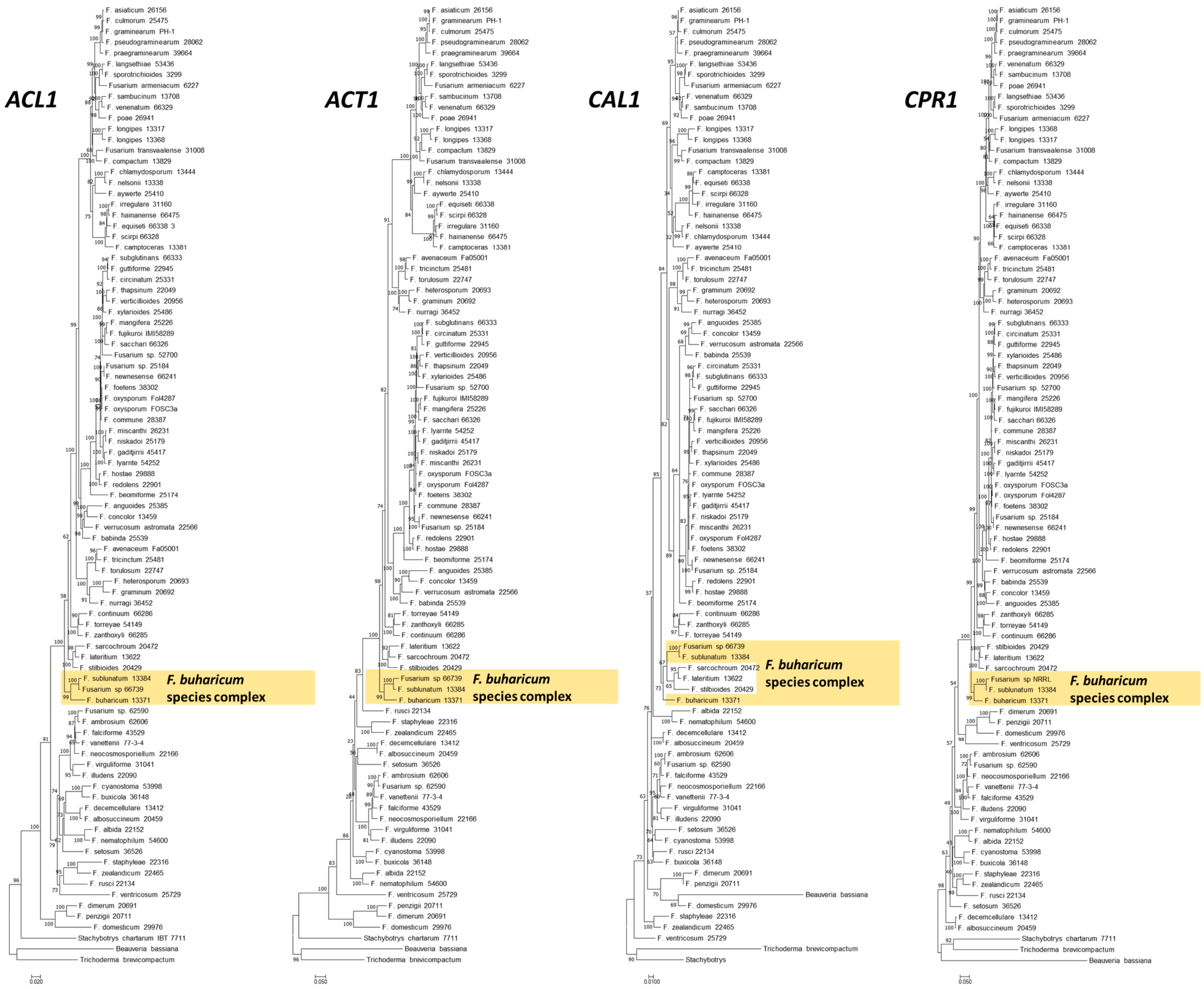

Supplementary Figure S1 continued

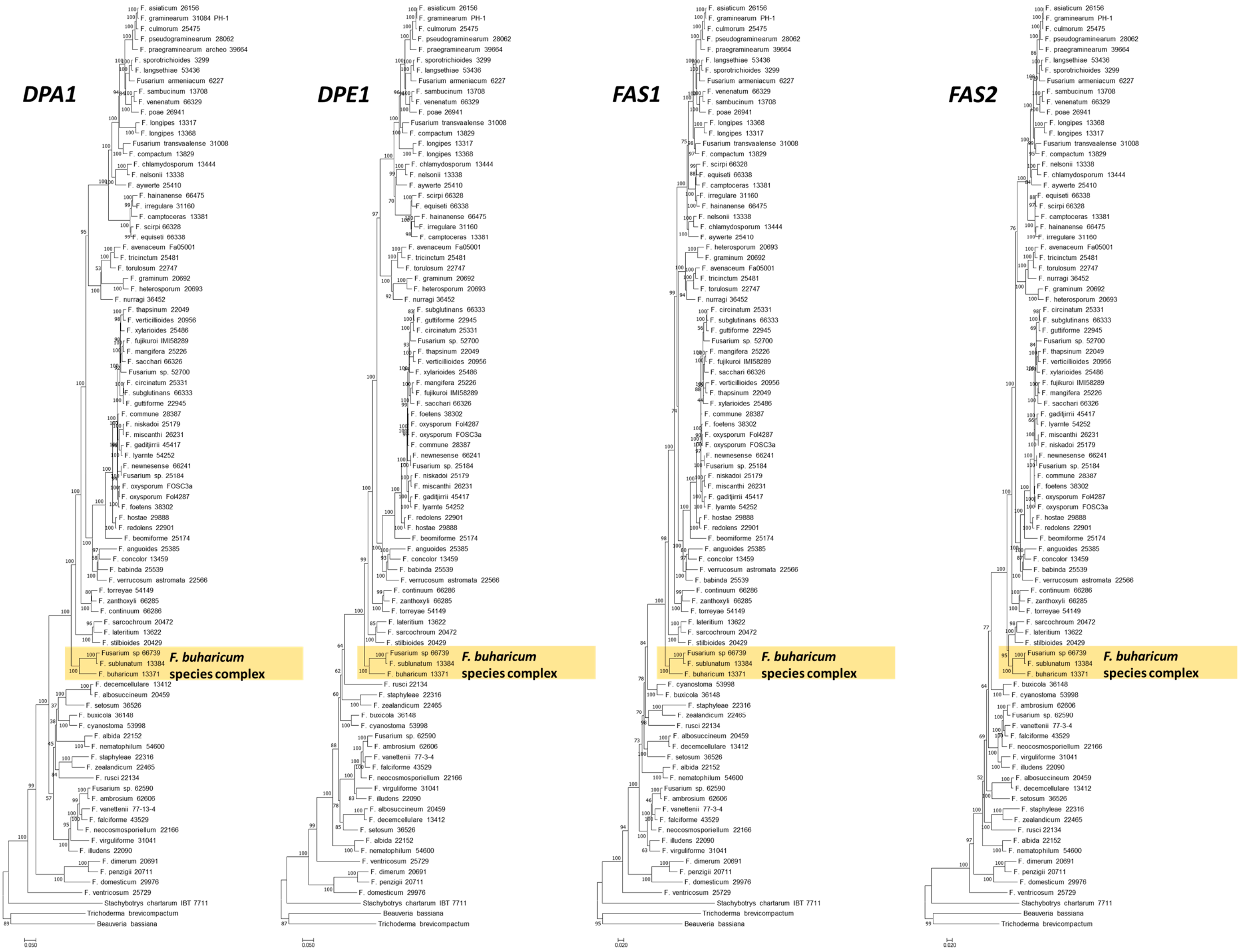

Supplementary Figure S1 continued

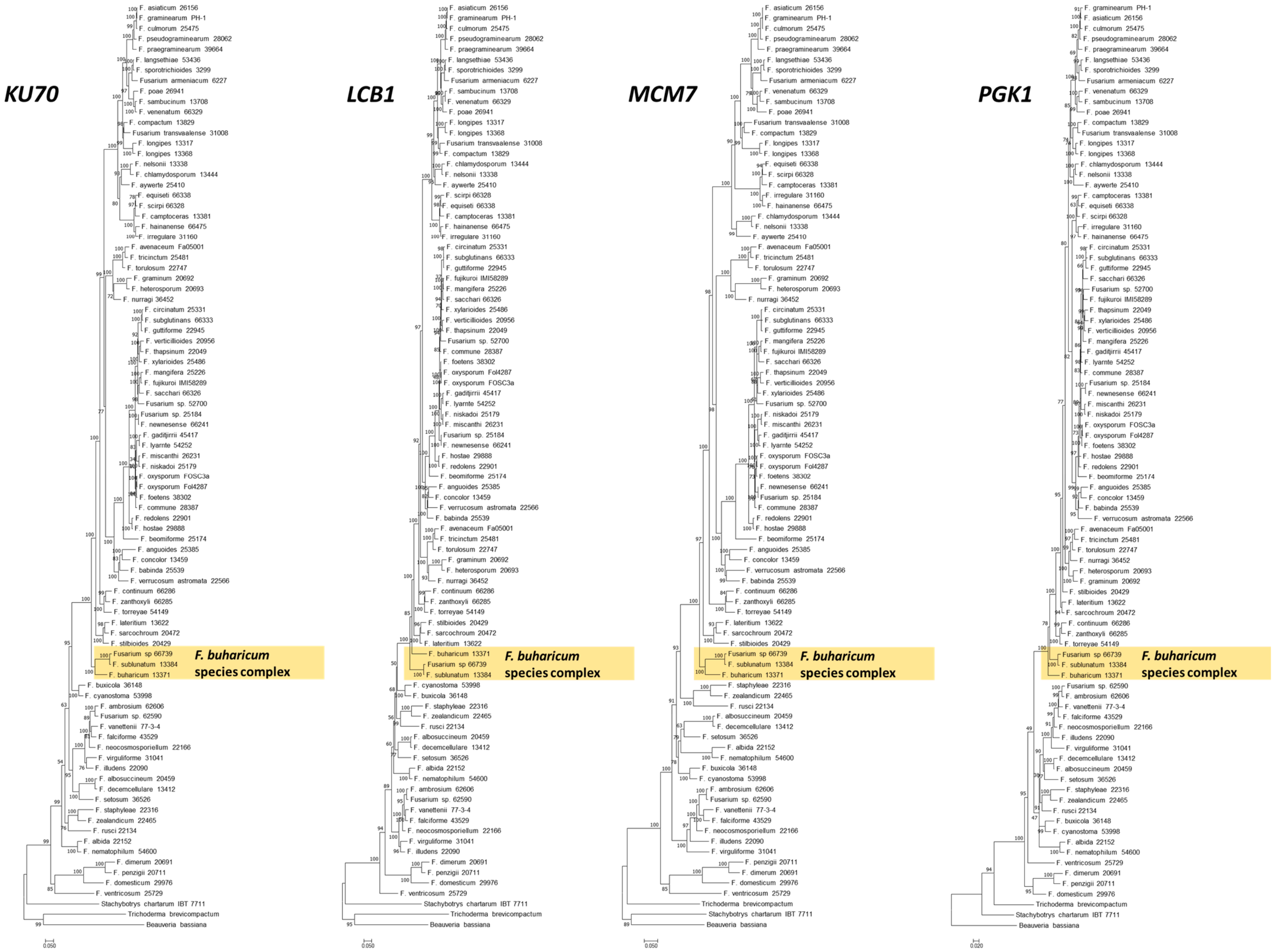

Supplementary Figure S1 continued

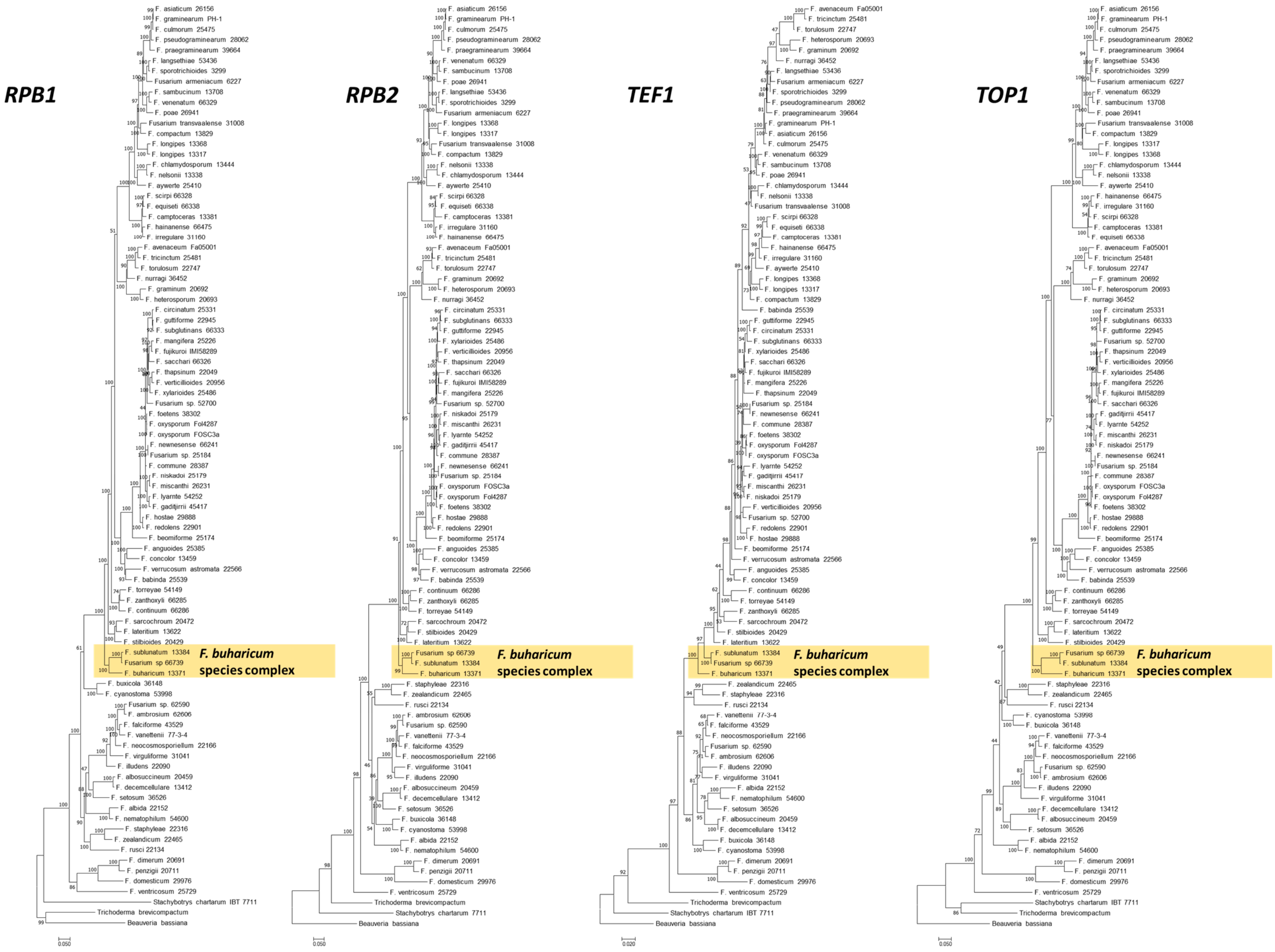

Supplementary Figure S1 continued

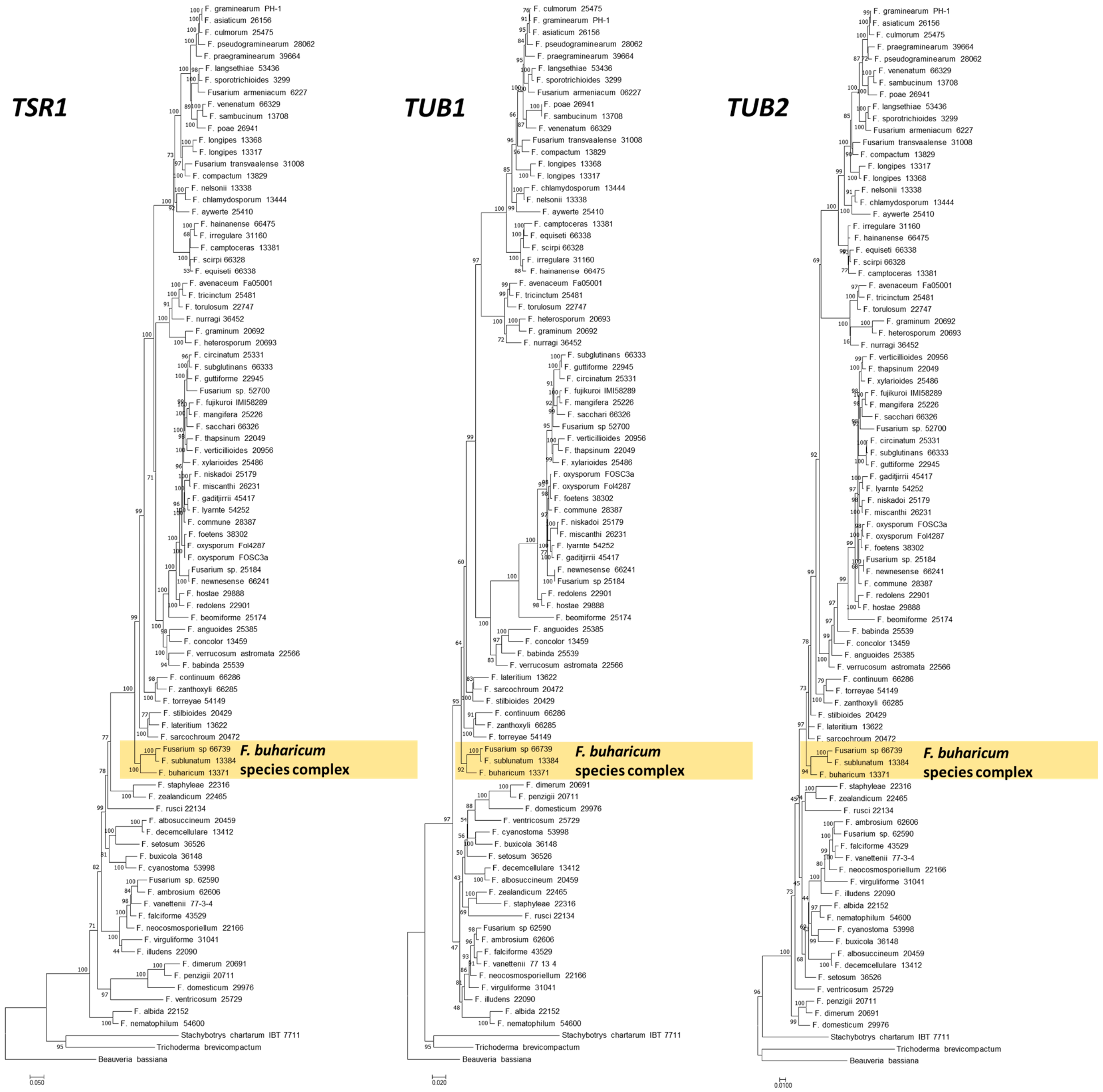

Figure S1. Individual gene trees for 19 housekeeping genes inferred by maximum likelihood analysis as implemented in IQ-Tree. Numbers near branches are bootstrap values based on 1000 pseudoreplicates using the ultrafast bootstrap parameter in IQ-Tree.

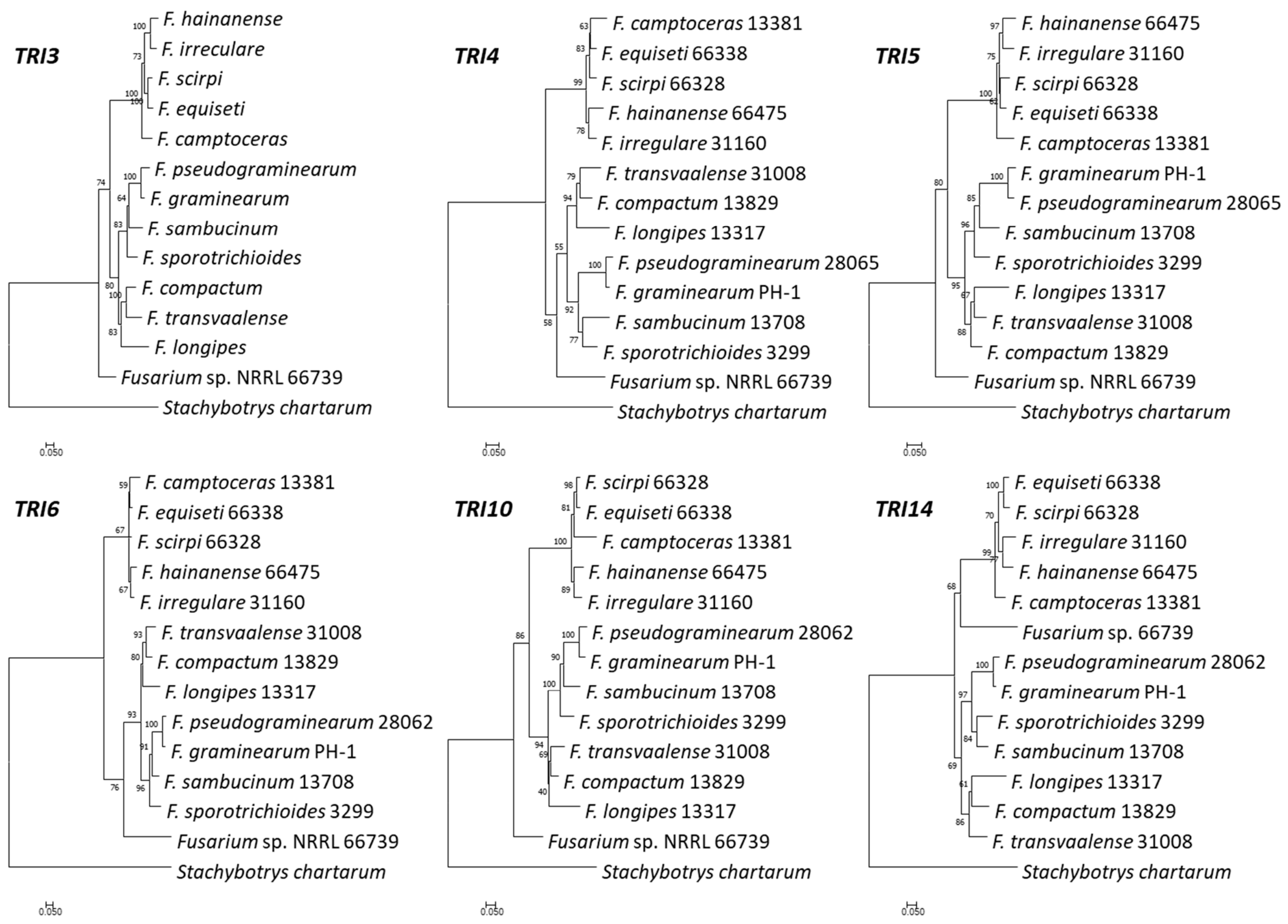

Figure S2. Individual gene trees for six trichothecene biosynthetic (*TRI*) genes inferred by maximum likelihood analysis as implemented in IQ-Tree. Numbers near branches are bootstrap values based on 1000 pseudoreplicates using the ultrafast bootstrap parameter in IQ-Tree.

Table S1. PCR primers used in this study.

Primer name	Sequence (5′ → 3′)[a]
TEF1-5-up	GGACTTAAUCCGCGGATCCAGCAAACGGT
TEF1-3	GTTTGACGGTTGTGTATGGAAGATTGAGTG
TEF1-664F	AAGCTTTGACCTCCTCGAGC
TRI13-5	ACACAACCGTCAAACATGATATTCATTTCATTCGCGCTGGCAC
TRI13-3-down	GGGTTTAAUTTATTCGATATAAAGACTAGTCTCTC
TRI13-399R	GCGGTGGGCTCTAAAGGTAG
CPM1-5	ACACAACCGTCAAACATGATATTCATTTCATTCGC
CPM1-3-down	GGGTTTAAUTCAGTTATGTAACTTGGTAAACTTGATCAG
CPM1-437R	AAATACCGCCAGACCTGTCG

[a] A single underline indicates sequence added to primers to facilitate USER cloning (New England Biolabs). A double underline indicates sequence complementary to the 3′ end of *AbTEF1*pro that were added to primers to facilitate fusion of *AbTEF1*pro with the *TRI13* or F1155_1930 coding regions.
